# Suppressing Primer-Driven Nonspecific Amplification in LAMP Using TrueLAMP

**DOI:** 10.1101/2025.10.23.684253

**Published:** 2026-04-27

**Authors:** Zhidong Chen

**Affiliations:** Boltii Diagnostics Inc., Salt Lake City, Utah, USA

**Keywords:** LAMP, colorimetric detection, nonspecific amplification, point-of-care diagnostics, TrueLAMP^™^

## Abstract

Loop-mediated isothermal amplification (LAMP) is widely used for rapid nucleic acid detection but is limited by primer-driven nonspecific amplification that produces false positives. We developed TrueLAMP, a colorimetric LAMP formulation containing an inhibitor that suppresses nonspecific amplification while preserving target-dependent reactions. Using SARS-CoV-2 as a model, TrueLAMP was evaluated across multiple primer sets. TrueLAMP eliminated amplification in no-template controls while maintaining robust target amplification, achieving 100% specificity across all no-template controls and, using the Lamb primer set as a representative benchmark, a limit of detection of 250 copies per reaction with 95% detection probability. Stable endpoint colorimetric signals were maintained over extended incubation periods, enabling flexible readout without time-critical monitoring. TrueLAMP is compatible with readily available equipment, including convection ovens and temperature-controlled smart mugs, supporting simplified and decentralized molecular testing workflows.

## METHOD SUMMARY

Colorimetric LAMP reactions targeting SARS-CoV-2 were performed using TrueLAMP polymerase and buffer containing a nonspecific amplification inhibitor. Reactions were carried out using a two-step incubation (60 °C for 10 min followed by 65 °C) in either a convection oven or a smart mug.

Amplification was monitored by colorimetric change and quantified using smartphone-based magenta or green channel intensity measurements. Orthogonal validation was performed by agarose gel electrophoresis using reactions with and without the inhibitor.

## 1. Introduction

Loop-mediated isothermal amplification (LAMP) has become a prominent method for rapid nucleic acid detection due to its high amplification efficiency and operation under isothermal conditions, making it ideal for point-of-care and field testing [1–4]. Despite its potential as a research and diagnostic tool, the widespread use of LAMP has been limited by a persistent problem: nonspecific amplification occurring even when no template DNA or RNA is present. Such spurious amplification leads to false positives in no-template controls (NTCs) and, potentially, test samples, undermining assay reliability [5–7].

When confronted with these false positives, researchers often suspect contamination and spend considerable time addressing it—replacing reagents and consumables, implementing separate preparation and reaction areas, or incorporating uracil-DNA glycosylase (UDG) and dUTP systems to prevent cross-contamination. When nonspecific amplification persists, practitioners frequently redesign and rescreen new primer sets. This recurring issue can hinder assay development and broader adoption.

Numerous additives, including betaine, DMSO, tetramethylammonium chloride (TMAC), and pullulan, have been explored to improve LAMP specificity, but none consistently prevent primer-driven amplification [7–10]. Many reports describe partial improvements based on short reaction times, yet when incubations extend beyond 40–60 minutes, nonspecific amplification typically reappears.

Several recent strategies have sought to overcome LAMP’s nonspecific amplification by coupling the reaction to sequence-specific nucleases such as CRISPR/Cas12a or programmable Argonaute (pAgo) proteins. In these hybrid systems, the nuclease is activated only when the correct target sequence is bound by a complementary guide RNA or DNA, thereby rejecting off-target or primer-derived amplicons and improving assay specificity [11–14]. While effective, such approaches introduce additional reagents, guide design steps, and detection layers, increasing assay complexity and cost.

We hypothesized that nonspecific amplification in LAMP is an intrinsic property of the reaction chemistry, arising from primer–primer interactions and secondary structures that can be extended by Bst-type polymerases. With their strong strand-displacement and low-fidelity activity, these polymerases can elongate short, mismatched primer duplexes and generate spurious amplicons. Mechanistic studies have confirmed this pathway, demonstrating that false-positive signals in NTCs result from the amplification of primer dimers, self-priming hairpins, or other mispriming events [15–17].

Here, we describe TrueLAMP, a polymerase formulation that includes a proprietary inhibitor selectively blocking nonspecific primer amplification without compromising target-specific amplification. Unlike conventional additives that provide only a brief window of inhibition—typically lasting minutes—TrueLAMP achieves durable suppression of primer-driven amplification over extended incubation periods. Furthermore, unlike LAMP-coupled CRISPR, pAgo, and other sequence-specific post-LAMP detection systems, TrueLAMP suppresses primer-derived nonspecific amplification directly within the amplification reaction, preserving the simplicity, accessibility, and low cost that make isothermal amplification attractive for point-of-care applications. We evaluated TrueLAMP across multiple primer sets and demonstrated its effectiveness in blocking false positives while maintaining robust amplification of target nucleic acids. By maintaining assay simplicity while eliminating false positives, TrueLAMP directly addresses the reliability barrier that has prevented LAMP from realizing its full potential in decentralized and low-resource testing environments.

## 2. Materials and methods

### 2.1. Reagents and Enzymes

TrueLAMP^™^ kits were obtained from Boltii Diagnostics Inc. (Salt Lake City, Utah), comprising TrueLAMP polymerase and 2× TrueLAMP buffer containing an inhibitor that suppresses nonspecific LAMP amplification. The inhibitor composition is proprietary and not disclosed. SARS-CoV-2 RNA (NR-52347) and heat-inactivated SARS-CoV-2 virus (NR-52286) were obtained from BEI Resources, NIAID, NIH.

### 2.2. Primer Sets

Four independent LAMP primer sets targeting the SARS-CoV-2 genomic RNA were used to assess the performance and generality of TrueLAMP: one proprietary set (N008) from Boltii Diagnostics and three published sets—’Lamb,’ ‘N27,’ and ‘M3.’ The Lamb primer set corresponds to the SARS-CoV-2 RT-LAMP design by Lamb et al. (2020) [18], and the N27 and M3 primer sets were adopted from the LAMP optimization study by Huang, Tang, Ismail, and Wang (2022) [19]. Full primer sequences are provided in Supplementary Table S1. All sequences used in this study match those reported in the original publications. Final primer concentrations in the 10 μL reaction were 0.2 μM (F3/B3), 0.4 μM (LF/LB), and 1.6 μM (FIP/BIP).

### 2.3. LAMP Reactions

Reactions were performed as described below, with additional procedural details provided in Supplementary Protocol S1. Each 10 μL reaction contained 3.0 μL nuclease-free water, 5.0 μL 2× buffer, 0.05 μL polymerase, 1.0 μL 10× primer mix, and either 1.0 μL nuclease-free water (no-template control, NTC) or 1.0 μL template (RNA, DNA, or virions). Reactions were incubated using a two-step protocol (60 °C for 10 min followed by 65 °C for 80 min). Positive reactions changed from red to orange/yellow, whereas NTCs remained red. Colorimetric signals were quantified by measuring either the maximum magenta (CMYK mode) or minimum green (RGB mode) intensity using a smartphone colorimeter app. Measurements were taken immediately below the liquid meniscus using the smallest available sampling aperture. Both metrics reflect the same underlying red-to-yellow color transition during amplification.

### 2.4. Colorimetric LAMP Setups

**Figure 1.**
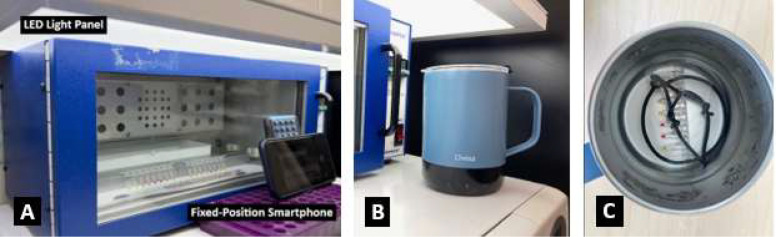
Semi-real-time and endpoint colorimetric LAMP setups using off-the-shelf equipment. (A) Semi-real-time monitoring using a convection oven and fixed-position smartphone imaging under consistent lighting conditions. (B) A low-cost heating device (temperature-controlled smart mug) used as an endpoint incubator. (C) Interior view of the smart mug showing immersion of 8-strip reaction tubes, which are weighed down to maintain consistent thermal contact. These configurations illustrate that TrueLAMP reactions can be performed using unmodified, widely available equipment.

Due to the small 10 μL reaction volume, uniform heating of reaction tubes from all directions is critical to minimize evaporation and condensation. Representative reaction setup and imaging configurations are shown in Figure 1. For semi-real-time LAMP, eight-strip PCR tubes were placed upright in a convection hybridization oven (Stratagene PersonalHyb, Model 401030). The 8-strip was photographed at 15 min intervals through the oven viewing window using a fixed-position smartphone. Maintaining constant imaging geometry and lighting was essential, as variations in angle, distance, or illumination can affect numerical color values. For endpoint LAMP, a laboratory water bath or a temperature-controlled smart mug was used as a simplified heating device. Reaction temperature was verified using a liquid thermometer or a thermocouple positioned at the tube location.

### 2.5. Statistical analysis

Quantitative colorimetric data were analyzed using descriptive statistics (mean ± standard deviation). Group comparisons of magenta intensity over time were performed using the parametric Welch’s t-test [20] and the non-parametric Mann–Whitney U test [21]. Differences were considered statistically significant at p < 0.05. For binary outcome data, including sensitivity and specificity assays, Fisher’s exact test [22] was used to assess categorical associations. Confidence intervals (95% CI) for proportions were calculated using the Clopper–Pearson exact method [23]. All analyses were performed using GraphPad Prism.

## 3. Results and discussion

### 3.1. Blocking Primer-Driven Nonspecific Amplification

**Figure 2.**
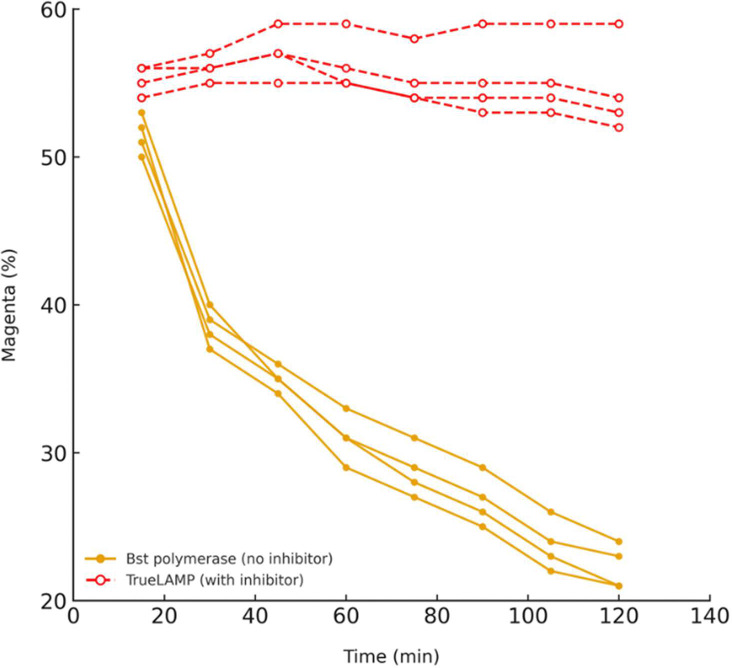
Suppression of primer-driven nonspecific amplification in no-template controls. Time-course colorimetric LAMP reactions were performed with the amplification-prone M3 primer set in the absence of template using reactions without inhibitor or with the TrueLAMP inhibitor. Inhibitor-free reactions showed progressive loss of magenta intensity consistent with nonspecific amplification, whereas inhibitor-containing reactions remained stable throughout incubation.

To evaluate whether TrueLAMP suppresses primer-driven nonspecific amplification, no-template control (NTC) reactions were performed using the amplification-prone M3 primer set under identical conditions with conventional Bst polymerase (without inhibitor) or TrueLAMP polymerase (with inhibitor) at 65 °C. Magenta intensity was monitored every 15 minutes for 120 minutes (Figure 2). In inhibitor-free reactions, magenta intensity decreased progressively from 51.5 ± 1.3 at 15 min to 22.3 ± 1.5 at 120 min, indicating progressive color loss consistent with nonspecific amplification. In contrast, TrueLAMP reactions maintained stable red color throughout incubation, with magenta intensities remaining between 55.3 ± 1.0 and 54.5 ± 3.2.

Statistical analysis confirmed significant differences between conditions at all time points (Welch’s t-test: p < 0.005 at 15 min; p < 0.0001 from 30 min onward), and a Mann–Whitney U test yielded consistent significance (p ≈ 0.0286). These results demonstrate that primer-driven amplification readily occurs in conventional LAMP but is effectively suppressed in TrueLAMP.

Primer-driven nonspecific amplification is a well-recognized limitation of LAMP, arising from primer–primer interactions and self-priming events that are extended by strand-displacing polymerases. The complete absence of color change in TrueLAMP NTCs indicates that this formulation directly suppresses these events, addressing a central limitation of the method without altering reaction conditions.

### 3.2. Orthogonal Validation of Amplification Products

**Figure 3.**
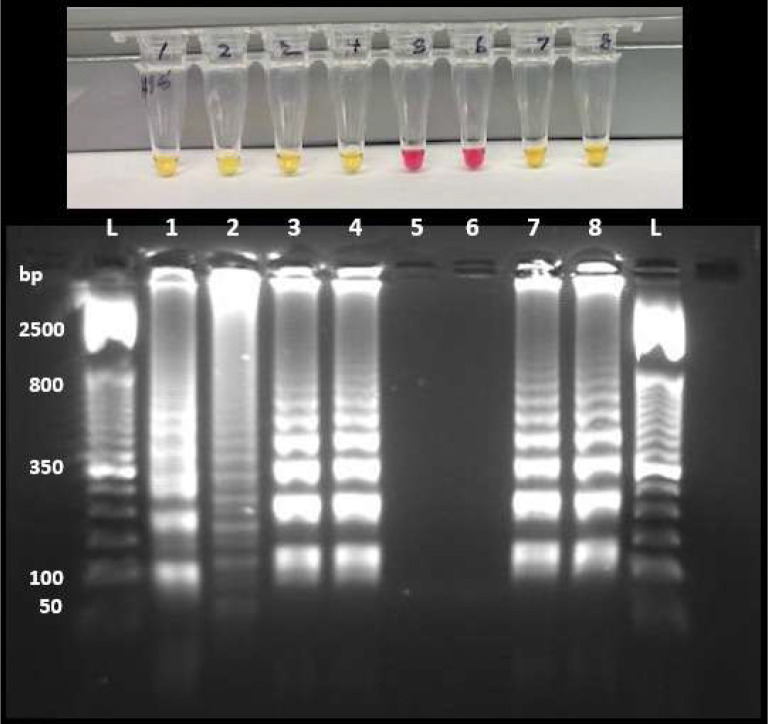
Orthogonal validation of inhibitor-dependent suppression of nonspecific amplification. Representative colorimetric reactions (top) and corresponding agarose gel analysis (bottom) performed using the same polymerase and colorimetric buffer background with or without the nonspecific amplification inhibitor (M3 primer set). In the absence of inhibitor (lanes 1–4), NTCs (lanes 1 and 2) showed color change and amplification products. In contrast, inhibitor-containing NTCs (lanes 5 and 6) remained red and showed no detectable amplification products. Template-containing reactions using SARS-CoV-2 heat-inactivated virions (NR-52286) amplified in both conditions (lanes 3 and 4 and lanes 7 and 8), confirming that the inhibitor suppresses nonspecific amplification without abolishing target-dependent amplification.

To confirm that the colorimetric changes correspond to nucleic acid amplification rather than indicator artifacts, LAMP amplification products were analyzed by agarose gel electrophoresis using the same polymerase and buffer background with or without the nonspecific amplification inhibitor (Figure 3). Reactions were performed using the M3 primer set with SARS-CoV-2 heat-inactivated virions (NR-52286) or NTCs in a 90-min endpoint RT-LAMP using a smart mug.

In inhibitor-free reactions, NTC samples exhibited both color change and amplification products, whereas inhibitor-containing NTCs showed no detectable amplification. Template-containing reactions produced characteristic LAMP amplification products in both conditions, indicating that target amplification was preserved.

Notably, amplification products observed in inhibitor-free NTC reactions displayed banding patterns distinct from those of template-derived products, consistent with nonspecific primer-derived amplification. These results confirm that the colorimetric signal directly reflects amplification and that TrueLAMP selectively suppresses nonspecific amplification without inhibiting target-dependent reactions, consistent with observations under convection oven incubation.

While the precise mechanism of inhibition is not disclosed, the selective suppression of NTC amplification with preservation of target amplification suggests preferential inhibition of primer-driven extension events rather than general suppression of polymerase activity. The inhibitor was identified empirically through screening rather than rational design. Notably, TrueLAMP polymerase loses activity within hours of being combined with the 2× buffer at any storage temperature, including at −20 °C, whereas the polymerase is stable when stored separately. This time-dependent inactivation upon mixing suggests that the inhibitor forms a progressive complex with the polymerase after assembly. We speculate that this interaction preferentially attenuates the low-fidelity, strand-displacement extension of short mismatched primer duplexes that underlies nonspecific amplification, while leaving sufficient processive activity for elongation on fully annealed, stable primer–template complexes during the reaction window. Further biochemical characterization will be needed to confirm this model.

### 3.3. Performance Across Multiple Primer Sets

**Figure 4.**
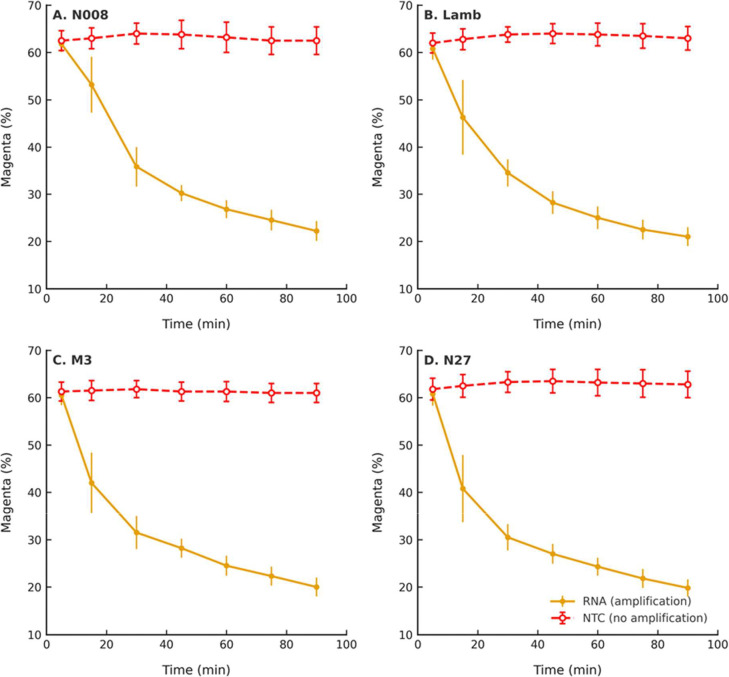
Suppression of primer-driven amplification across multiple primer sets. Semi-real-time colorimetric LAMP reactions performed with four primer sets (N008, Lamb, M3, and N27). Template-containing reactions (solid lines) showed robust amplification, while no-template controls (dashed lines) remained stable in TrueLAMP reactions. Results demonstrate suppression of nonspecific amplification across multiple tested primer sets.

To assess generality, semi-real-time colorimetric LAMP amplification of SARS-CoV-2 RNA (500 copies) and NTCs was evaluated using four independent primer sets (N008, Lamb, M3, and N27) across replicate experiments (Figure 4). These sets were selected to represent varying amplification characteristics, including primer sets prone to nonspecific amplification, providing a stress test for the system.

Across all primer sets, NTC reactions maintained stable magenta intensity throughout the 90-minute incubation period, confirming suppression of nonspecific amplification. In contrast, template-containing reactions exhibited rapid decreases in magenta intensity beginning at approximately 15 minutes, when a visible color shift became apparent, and exceeding 50% reduction by 30 minutes.

Statistical analysis confirmed significant separation between template and NTC reactions (Welch’s t-test: p < 0.05 at 15 min and p < 0.0001 after 30 min for all primer sets; Mann–Whitney U test: consistent significance after 30 min). These results demonstrate that TrueLAMP amplifies target nucleic acids while suppressing background amplification across multiple primer designs.

While primer design remains a key determinant of LAMP performance, the consistent absence of NTC amplification across these independently designed primer sets indicates that TrueLAMP reduces primer-driven artifacts across the tested designs. Similar suppression has been observed during internal evaluation of over 20 additional primer sets developed in the context of multiplex assay development for human papillomavirus (HPV) detection (data not shown).

### 3.4. Analytical Sensitivity

Analytical sensitivity was evaluated using serial dilutions (1000–16 copies/μL) of SARS-CoV-2 RNA (NR-52347) amplified with the Lamb primer set in a convection oven. Reactions containing ≥250 copies per reaction consistently produced positive amplification, whereas lower concentrations yielded inconsistent detection.

**Figure 5.**
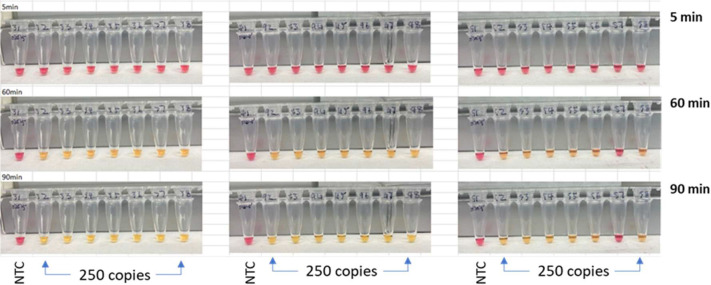
Confirmation of limit of detection (LOD) of TrueLAMP using the Lamb primer set. During the estimation of LOD, reactions containing ≥250 copies consistently produced positive amplification, while yielding inconsistent detection at lower concentrations. Triplicate 8-strip (n=21) confirmations showed 20/21 positive reactions (95% detection probability; 95% CI 77–99%), establishing a LOD of 250 copies per reaction.

LOD confirmation experiments (Figure 5) using three independent 8-strip reactions demonstrated 20 of 21 positive detections (95%) at 250 copies, with all NTCs remaining negative. The resulting detection probability (95%; 95% CI: 77–99%) establishes 250 copies per reaction as the LOD for the Lamb primer set under these conditions. This sensitivity is comparable to previously reported colorimetric LAMP assays using SARS-CoV-2 RNA as the template [24–26], indicating that the inhibitory component does not compromise analytical sensitivity.

A direct head-to-head LOD comparison between TrueLAMP and uninhibited LAMP was not performed, because extended incubation of uninhibited NTCs produces false-positive signals that confound sensitivity measurements at matched timepoints — a limitation that is itself the central motivation for this work. Meaningful comparison would require truncating uninhibited reactions to the narrow pre-false-positive window (typically less than 30 min), which would penalize the uninhibited condition by excluding the additional sensitivity gained from longer incubation. That the inhibitor does not impair target amplification is further supported by the consistent amplification kinetics observed across all four primer sets in Section 3.3 (Figure 4).

### 3.5. Two-Step Incubation Ensures Consistent Performance Across Targets, Primer Sets, and Heating Devices

Rapid equilibration to the amplification temperature can prevent proper primer annealing and amplification initiation for some primer sets. This was most clearly demonstrated using a 65 °C water bath (Fisher Scientific Model 2LS, Pittsburgh, PA), which equilibrates faster than convection ovens and served as a useful stress test: reactions showed reduced amplification efficiency and an elevated apparent LOD (>1000 copies), with only 14 of 21 reactions (67%) positive at 1000 copies, compared to 95% detection at 250 copies with convection oven incubation (Table 1).

Implementing a two-step incubation profile — 60 °C for 10 min followed by 65 °C — restored amplification efficiency, yielding 21 of 21 positive detections (100%) at 250 copies in the water bath. Importantly, the same failure mode — complete loss of amplification with direct 65 °C incubation, rescued by the two-step protocol — was also observed with DNA LAMP primer sets where reverse transcriptase is not involved. This indicates that the initial low-temperature phase provides a window for primer annealing and amplification initiation in TrueLAMP before the reaction transitions to the optimal amplification temperature.

The two-step protocol is therefore recommended as the universal standard incubation procedure, as it ensures consistent amplification initiation regardless of target type, primer set, or heating device — simplifying assay development and deployment without requiring optimization for individual applications. Importantly, TrueLAMP’s suppression of nonspecific amplification was maintained under all incubation conditions tested, confirming that the inhibitor’s selectivity is equally robust across heating modalities.

## 4. Conclusion

TrueLAMP addresses a central limitation of LAMP by directly suppressing primer-driven nonspecific amplification within the reaction. This selectivity is achieved without altering reaction conditions or compromising analytical sensitivity. Across multiple primer sets, NTCs remained stable while target amplification was preserved, and orthogonal gel analysis confirmed the absence of nonspecific amplification products. Stable endpoint signals over extended incubation periods enable flexible readout without time-critical monitoring. Unlike conventional LAMP, where late nonspecific amplification necessitates strict endpoint timing or differential time to threshold (DTT) analysis, TrueLAMP’s stable NTCs make result timing flexible and DTT unnecessary. Combined with compatibility with readily available equipment, including convection ovens and smart mugs, TrueLAMP preserves the simplicity of LAMP while enhancing its reliability. Together, these features support the practical usability of LAMP for routine, decentralized, and resource-limited molecular testing applications.

## 5. Future perspective

This study evaluated TrueLAMP using a limited number of primer sets and targets under controlled laboratory conditions. All experiments were conducted by the inventor within the developing company; independent replication by external laboratories will be important to confirm the generalizability of these findings and to establish confidence appropriate for widespread adoption. Broader validation across additional primer designs, nucleic acid targets, and sample types will help establish general applicability. Further benchmarking against alternative specificity-enhancing approaches such as probe-based detection and CRISPR-assisted systems will help define the relative advantages and limitations of this approach. Evaluation using clinical and environmental samples will further define real-world performance and support potential regulatory translation. Future work should also focus on deployment in simplified and field-compatible workflows. This includes assessment in portable or battery-powered heating systems and integration with low-cost sample preparation and smartphone-based result interpretation. Such developments would enable complete, instrument-minimal molecular testing workflows and expand the applicability of TrueLAMP across clinical, public health, veterinary, and environmental settings.

## Supplementary Material

Supplement 1

## Figures and Tables

**Table 1.** Effect of incubation profile on RT-LAMP limit of detection (LOD)*.

Incubation condition	RNA (copies)	Positive detections	95% CI (exact)	LOD (copies)	*p*-Value (vs *p*_0_ = 0.95)

1. Oven (65 °C)	250	20/21 (95%)	77–99%	250	0.66 (pass)
2. Water bath (65 °C)	1000	14/21 (67%)	43–85%	> 1000	4.9 × 10^−⁵^ (fail)
3. Water bath (60→65 °C)	250	21/21 (100%)	84–100%	250	> 0.99 (pass)

* Twenty-one replicates were tested per condition with 3 no-template controls (all negative) using the Lamb primer set. LOD defined as concentration giving ≥ 95% detection (≥ 20 / 21 positives).

## Data Availability

All data supporting the findings of this study are included within the article and its Supplementary Materials. The datasets used and/or analyzed during the current study are available from the corresponding author upon reasonable request.

## HIGHLIGHTS

- TrueLAMP completely suppresses primer-driven nonspecific amplification in no-template controls across multiple primer sets.
- TrueLAMP achieved 100% specificity across all no-template controls and a limit of detection of 250 RNA copies per reaction with 95% detection probability (95% CI: 77–99%) using the Lamb primer set.
- Agarose gel analysis confirmed elimination of nonspecific amplification products while preserving target-dependent amplification.
- A two-step (60 °C → 65 °C) incubation ensured consistent amplification across different primer sets and heating modalities.
- Stable endpoint colorimetric signals enable flexible assay readout without time-critical monitoring.
- The method maintains LAMP simplicity while improving reliability for routine and decentralized testing applications.
