# Supplementary material for: Suppressing Primer-Driven Nonspecific Amplification in LAMP Using TrueLAMP
Source: bioRxiv. 2026 Apr 27:2025.10.23.684253. Preprint. [Version 4] doi: 10.1101/2025.10.23.684253 (PMC13142356; doi:10.1101/2025.10.23.684253)
Supplement: Supplement 1 [file media-1.pdf]

**Supplementary Table S1. Primer sequences used in this study**

| Set | Name | Sequence (5′ → 3′) | Target | Reference |
|---|---|---|---|---|
| **Lamb** | F3 | TCCAGATGAGGATGAAGAAGA | **ORF1ab** | Lamb et al., 2020 |
| | B3 | AGTCTGAACAACTGGTGTAAG | **ORF1ab** | Lamb et al., 2020 |
| | FIP | AGAGCAGCAGAAGTGGCACAGGTGATTGTGAAGAAGAAGAG | **ORF1ab** | Lamb et al., 2020 |
| | BIP | TCAACCTGAAGAAGAGCAAGAACTGATTGTCCTCACTGCC | **ORF1ab** | Lamb et al., 2020 |
| | LF | CTCATATTGAGTTGATGGCTCA | **ORF1ab** | Lamb et al., 2020 |
| | LB | ACAAACTGTTGGTCAACAAGAC | **ORF1ab** | Lamb et al., 2020 |
| **N27** | F3 | CCCTCAGATTCAACTGGCAGTA | **N** | Huang et al., 2022 |
| | B3 | TTCATTTTACCGTCACCACCAC | **N** | Huang et al., 2022 |
| | FIP | GAGAGCGGTGAACGGGCGCGATCAAAACAACG | **N** | Huang et al., 2022 |
| | BIP | TCGAGGACAAGGCTTCGGTAGTAGCCAATTTGGTC | **N** | Huang et al., 2022 |
| | FL | GACGCAGTATTATTGGGTAAACC | **N** | Huang et al., 2022 |
| | BL | CCAATTAACACCAATAGCAGTCCA | **N** | Huang et al., 2022 |
| **M3** | F3 | GCTATCGCAATGGCTTGTCTTG | **M** | Huang et al., 2022 |

| Set | Name | Sequence (5′ → 3′) | Target | Reference |
|---|---|---|---|---|
| | B3 | GAAAGCGTTCGTGATGTAGCAA | **M** | Huang et al., 2022 |
| | FIP | CAGAATAGTGCCATGCCATGTGGTCATTCAATCCAGA | **M** | Huang et al., 2022 |
| | BIP | AGAAAGTGAACTCGTCGTCCTAGATGGTGTCCAGCAA | **M** | Huang et al., 2022 |
| | FL | GGCACGTTGAGAAGAATGTTAGT | **M** | Huang et al., 2022 |
| | BL | GATCCTTCGTGGACATCTTCGT | **M** | Huang et al., 2022 |
| **N008** | F3 | *Proprietary* | **N** | Boltii Diagnostics Inc. |
| | B3 | *Proprietary* | **N** | Boltii Diagnostics Inc. |
| | FIP | *Proprietary* | **N** | Boltii Diagnostics Inc. |
| | BIP | *Proprietary* | **N** | Boltii Diagnostics Inc. |
| | LF | *Proprietary* | **N** | Boltii Diagnostics Inc. |
| | LB | *Proprietary* | **N** | Boltii Diagnostics Inc. |

**Footnote:** The **Lamb** primer sequences for published SARS-CoV-2 LAMP assays were obtained from *Lamb et al.* (2020, *medRxiv* 2020.02.26.20028373) and the **M3** and **N27** primer sequences were from *Huang et al.* (2022, *EBioMedicine* 75: 103736). The **N008** set was designed by Boltii Diagnostics Inc.; sequences are proprietary and not disclosed.

**Supplementary Protocol S1. TrueLAMP Protocol**

**Overview**

This protocol describes the setup, incubation, and detection of colorimetric loop-mediated isothermal amplification (LAMP) reactions using the TrueLAMP polymerase and buffer system. The formulation includes an inhibitor designed to suppress primer-driven nonspecific amplification while preserving target-dependent amplification. Guidance for incubation conditions and troubleshooting is provided to support reproducibility.

**Materials and Reagents**

- TrueLAMP polymerase
- 2× TrueLAMP buffer
- 10× LAMP primer mix (see Supplementary Table S1)
- Template nucleic acid (RNA or DNA)
- Nuclease-free water
- 0.2 mL 8-strip PCR tubes

**Reaction Setup (10 µL)**

- 2× buffer: 5.0 µL
- 10× primer mix: 1.0 µL
- Polymerase: 0.05 µL
- Template or NTC: 1.0 µL
- Water: 3.0 µL

**Procedure**

1. Prepare master mix excluding template.
2. Dispense 9 µL into each tube.
3. Add 1 µL template or water (NTC).
4. Mix and centrifuge briefly.

**Incubation Conditions**

60 °C for 10 min, followed by 65 °C for 50–80 min.

**Detection**

Positive reactions: yellow/orange. NTC: red.

**Frequently Asked Questions**

1. Should the kit be stored at -20 °C?

Store polymerase at -20 °C and 2x buffer at 22 °C. Do not vortex to resuspend the precipitates if developed during storage.

2. What incubator works best?

Uniform heating is critical to avoid evaporation and condensation in 10 µL reactions. Recommended: Fully submerged water bath (best for multiple reaction strips) or temperature-controlled smart coffee mug (POC) or thermocycler with heated lid. Convection oven may also work. Do not use heat block.

3. Why is my NTC positive?

1) Recalibrate pipettes. Incorrect volumes (< 4.75 µL buffer per 10 µL reaction) may reduce inhibition. 2) Verify incubation temperature. Nonspecific amplification can occur at ⩽ 63 °C after ~30 min. 3) Increase buffer slightly (e.g., 5.5 µL 2× buffer per 10 µL reaction) for stubborn primer sets.

4. Why did my positive control fail?

Template concentration may be below LOD. Determine LOD for each primer set.

5. Can raw samples (e.g., saliva, plasma) be used?

Purified DNA/RNA is recommended. TrueLAMP is sensitive to pH and ionic strength. Water-suspended cells, bacteria or virions may be compatible after validation.

6. Can polymerase be premixed with 2× buffer for storage?

No. The polymerase and 2× buffer must be stored as separate components. Once combined, polymerase activity declines gradually as it binds to the inhibitor in the buffer, and this effect is irreversible. Prepare the master mix fresh and proceed to amplification within 30 minutes of assembly.

7. Why is a 60 °C pre-incubation needed and how long should reactions run?

An initial 58-61 °C 10 min pre-incubation followed by 65 °C for 50-80 min improves amplification consistency across DNA and RNA targets, primer sets, and incubation systems (oven, bath, thermocycler). Amplification is generally visible by 30 min and reaches maximum by 90 min. Strict endpoint timing is not required. Extended incubation does not typically alter endpoint color.

8. How should color be quantified?

Use a smartphone colorimeter app. Sample the region just below the liquid meniscus with the smallest aperture and record: minimum green (RGB mode) or maximum magenta (CMYK mode).

9. Do I need to determine differential time to threshold (DTT)?

No. Because NTCs remain reliably negative regardless of incubation duration, result interpretation does not depend on when the reaction is read. A simple visual or smartphone color comparison at any convenient endpoint is sufficient. This eliminates the need for DTT analysis that conventional LAMP requires to avoid false-positive interference.
